# Improving Patient Throughput in a Busy Pediatric Mri Imaging Program Through a Nursing Coordinator

**DOI:** 10.1186/1532-429X-18-S1-P309

**Published:** 2016-01-27

**Authors:** Sanket Shah, Martha Carter, Joshua Q Knowlton

**Affiliations:** 1 Cardiology, CMH, Kansas City, MO USA; 2 Radiology, Children's Mercy Hospital, Kansas City, MO USA

## Background

Pediatric cardiac MRIs form a small but critical part of MRI imaging at a busy pediatric cardiac imaging center. We had a higher than acceptable rate of no-show and wait times for scheduled cardiac MRIs, which was a source of patient and provider dissatisfaction in our institution. Previously, the process of scheduling a cardiac MRI was facilitated by a nonmedical provider. Dedicated nurse coordinator was added to the Pediatric Cardiac MRI scheduling team to provide a centralized and focused source for referral intakes and coordination of care.

## Methods

The nursing coordinator facilitates scheduling and communication with patients and referring providers. Providers were given a direct phone number and primary fax number to provide ease of referrals and consultations. Patients were given reminder phone calls, emails and letters. During this communication, information about expectations for the MRI visit, directions, overnight accommodation at Ronald McDonald House, transportation resources, NPO instructions were provided as and when necessary.

## Results

Between August 2013 and July 2014, 128 cardiac MRIs were performed by the cardiac imaging center and 11 patients did not report for MRI testing [no-show rate of about 9%]. Between August 2014 to July, 2015, 169 cardiac MRI have been performed at our hospital with only 6 patients [3.5%] not showing up for appointment.

## Conclusions

The addition of a nurse coordinator in a Pediatric Cardiac Imaging Center has resulted in reduced no-show rates. This in turn has resulted in reduced wait times for obtaining a pediatric cardiac MRI and efficient use of resources. A similar scheduling model could be considered where cardiac MRI scanner time and other resources are limited.

